# Supplementary material for: Sorghum (Sorghum bicolor L. Moench) Gluten-Free Bread: The Effect of Milling Conditions on the Technological Properties and In Vitro Bioaccessibility of Polyphenols and Minerals
Source: Foods. 2023 Aug 12;12(16):3030. doi: 10.3390/foods12163030 (PMC10453239; doi:10.3390/foods12163030)
Supplement: Supplementary file 1 [file foods-12-03030-s001.zip › foods-2529699-supplementary.pdf]

Sorghum (*Sorghum bicolor* L. Moench) Gluten-Free Bread: the Effect of Milling Conditions on the Technological Properties and *In Vitro* Bioaccessibility of Polyphenols and Minerals

María Isabel Curti [1,2], Pablo Martín Palavecino [3,4], Marianela Savio [1,2], María Verónica Baroni [3,5] and Pablo Daniel Ribotta [3,4,*]

1 Facultad Ciencias Exactas y Naturales, Universidad Nacional de La Pampa, Santa Rosa 6300, Argentina; mariacurti@gmail.com (M.I.C.);
msavio@exactas.unlpam.edu.ar (M.S.)
2 Instituto de Ciencias de la Tierra y Ambientales de La Pampa (INCITAP, CONICET -UNLPAM), Santa Rosa 6300, Argentina
3 Instituto de Ciencia y Tecnología de Alimentos Córdoba (ICYTAC, CONICET-UNC), Córdoba, 5000, Argentina;
pmpalavecino@gmail.com (P.M.P.); vbaroni@unc.edu.ar (M.V.B.)
4 Facultad de Ciencias Exactas, Físicas y Naturales, Universidad Nacional de Córdoba, Córdoba 5000, Argentina
5 Facultad de Ciencias Químicas, Departamento de Química Orgánica, Universidad Nacional de Córdoba, Córdoba 5000, Argentina
* Correspondence: pdribotta@unc.edu.ar. Tel.: +54-351-5353755 (ext. 17141)

Table S1. Particle size distribution parameters of sorghum flour

Sample	D 10 (μm)	D 50 (μm)	D90 (μm)	Av (D4,3) (μm)	Span
WWS-IM	30.3 [a]	437.8 [a]	786.6 [a]	431.6 [a]	1.9 [b]
PWS-IM	187.6 [b]	564.7 [a]	883.6 [b]	561.6 [b]	1.2 [a]
WS-RM	156.3 [b]	456.7 [a]	777.8 [a]	468.7 [ab]	1.4 [a]

Values are expressed as mean (n = 3). Different letters in the same column express significant differences ($p<0.05$). WWS: whole white sorghum flour; PWS: polished white sorghum flour; IM: impact milling; RM: roller milling. D4.3: volume mean diameter; D10, D50, D90: diameters where 10%, 50%, and 90% of the particle population are below these values, respectively. Span: polydispersity index.

Table S2. Polyphenolic compounds identified in sorghum flour and bread

Nº	Tr (min)	Tentative ID	Molecular formula	[M-H]- experimental (m/z)	[M-H]- calculated (m/z)	Fragments MS/MS (m/z)
1	12.1	Dicaffeoyl spermidine I	$C_{25}H_{30}N_3O_6$	468.2107	468.2140	332, 306
2	12.5	Dicaffeoyl spermidine II	$C_{25}H_{30}N_3O_6$	468.2210	468.2140	332, 306
3	12.7	Caffeoil ferroyl spermidine	$C_{26}H_{33}N_3O_6$	482.2274	482.2297	332, 306
4	14.1	2-O caffeoil glycerol	$C_{12}H_{13}O_6$	253.0736	253.0718	179, 161
5	15.3	Caffeic acid	$C_9H_7O_4$	179.0331	179.0350	
6	16.8	Coumaroil glycerol	$C_{12}H_{13}O_5$	237.0778	237.0768	
7	22.7	Dicaffeoil glycerol	$C_{21}H_{19}O_9$	415.1058	415.1032	253, 179, 161
8	24.4	Coumaroil cafeoil glycerol	$C_{21}H_{20}O_8$	399.1080	399.1085	253, 179, 235
9	24.6	Feruroyl caffeoil glycerol	$C_{22}H_{21}O9$	429.1174	429.1191	193, 235
10	25.4	Naringenin	$C_{15}H_{12}O_5$	271.0590	271.0612	151, 177
11	26.1	Luteolin	$C_{15}H_{10}O_6$	285.0379	285.0405	
12	26.5	Coumaroil-feruloyl-glycerol	$C_{22}H_{22}O_8$	413.1208	413.1242	193, 285
13	29.1	Apigenin	$C_{15}H_9O_5$	269.0435	269.0455	225
14	29.5	Hispidulin	$C_{16}H_{12}O_6$	299.0563	299.0561	284
15	31.4	Tri-hydroxy-octadecenoic acid	$C_{18}H_{33}O_5$	329.2315	329.2333	211, 229

Tr: retention time
